# Bridging the methodological gap in multitrauma literature: a competency-based educational framework and clinical appraisal filter

**DOI:** 10.55730/1300-0144.6208

**Published:** 2026-01-30

**Authors:** Şakir Hakan AKSU, Burcu DOĞAN, Merve YAZLA

**Affiliations:** 1 Department of Emergency Medicine, İzmir City Hospital, İzmir, Turkiye; 2 Department of Emergency Medicine, Ankara Etlik City Hospital, Ankara, Turkiye

**Keywords:** Bibliometrics, biostatistics, emergency medicine, evidence-based medicine, multiple trauma, statistics

## Abstract

**Background/aim:**

The statistical complexity of contemporary multitrauma literature has increased considerably in recent years, which may pose challenges for clinicians seeking to interpret high-impact research and apply evidence-based practices. Characterizing this methodological landscape and exploring education-oriented approaches may help to improve the interpretability of the literature and support informed clinical decision-making.

**Materials and methods:**

A cross-sectional analysis was conducted on the 100 most-cited multitrauma studies published in Science Citation Index Expanded journals indexed in the Web of Science database between January 2018 and November 2025. The statistical methods used in each article were categorized into the three competency levels of introductory, intermediate, and advanced based on a literature-derived classification framework. Associations between methodological characteristics and the journal’s impact factor (IF) were examined.

**Results:**

Across the 100 studies, a total of 1210 statistical procedures were identified, with a mean of 12.1 ± 3.8 methods per article. Only 8% (n = 8) of the studies could be fully interpreted using introductory-level statistical knowledge, whereas 92% required intermediate (n = 61) or advanced (n = 31) analytical competency. Articles employing advanced statistical methods (mean IF: 9.03), techniques for handling missing data (mean IF: 8.22), and randomized controlled trial designs (mean IF: 18.87) were published in significantly higher-impact journals (p < 0.05).

**Conclusion:**

The findings of this study suggest the presence of a methodological gap between high-impact multitrauma literature and the level of statistical training commonly received by clinicians. This gap may complicate the critical appraisal of contemporary research and the translation of evidence into practice. As a potential response, we propose a complementary dual strategy: a practical “methodological appraisal filter” to support current clinicians and a structured, competency-based biostatistics curriculum emphasizing regression modeling, survival analysis, and advanced epidemiological methods.

## 1. Introduction

Statistical methods in the medical literature have become increasingly complex in recent years [1,2]. In trauma research, this trend is particularly pronounced due to the inherently complex structure of multitrauma data, including heterogeneous injury patterns, time-dependent interventions, and multiple confounding factors. As a result, a growing gap may exist between the statistical expertise required to critically appraise high-impact trauma studies and the training typically provided during standard medical education.

This gap has important implications for evidence-based practice. Limited methodological literacy may hinder clinicians’ ability to accurately interpret complex analyses, potentially affecting the integration of scientific evidence into clinical decision-making [3–5]. Previous studies in other surgical fields have shown that a substantial proportion of the literature requires advanced statistical knowledge [1,6]. However, the scope and complexity of statistical methods used specifically in the multitrauma literature have not yet been systematically evaluated.

Therefore, the aim of this study was to comprehensively assess the diversity and complexity of statistical methods used in high-impact multitrauma research, to classify these methods according to the level of statistical training required for full interpretability, and to examine the relationship between methodological complexity and publication impact.

## 2. Materials and methods

### 2.1. Study design

This study employed cross-sectional methodological analysis to examine the diversity and frequency of statistical methods used in the adult multitrauma literature.

### 2.2. Data source and search strategy

A systematic literature search was conducted using the Web of Science (WoS) Core Collection database. The search scope included articles published between January 2018 and November 2025. This time period was selected to represent the literature published after the release of the 10th edition of Advanced Trauma Life Support (ATLS), updated in 2018 [7].

The search strategy was based on predefined keyword combinations containing trauma terminology and emergency department concepts. The search algorithm was constructed as follows: TS=((“polytrauma” OR “multi-trauma” OR “multiple trauma” OR “multiple injuries” OR “severe trauma” OR “major trauma” OR “high-energy trauma”) AND (“emergency department” OR “emergency medicine” OR “emergency service, hospital” OR “emergency room” OR “accident and emergency” OR “ED” OR “A&E” OR “acute care”)) AND PY=(2018–2026) AND LA=(English) AND DT=(Article) AND WC=(Emergency Medicine).

All articles were independently reviewed by two emergency medicine specialists. Discrepancies in methodological classification and statistical method categorization were resolved through consensus discussion, with adjudication by a third reviewer with formal training in biostatistics when required. For each study, the statistical methods used, the statistical analysis software (IBM SPSS Statistics, RStudio, Stata, etc.), the citation count, and the journal’s impact factor (IF) and quartile (Q1–Q4) for the corresponding publication year were recorded. Citation counts were obtained from the WOS Core Collection database, and values current as of the search completion date (November 2025) were used. Journal IFs and quartile rankings were determined based on Journal Citation Reports data for the year in which the article was published.

### 2.3. Inclusion and exclusion criteria

Only full-text, original English-language research articles published in journals indexed by the Science Citation Index Expanded (SCI-E) were selected using WoS database filters. Inclusion criteria were as follows: (i) studies conducted with an adult population aged ≥18 years, (ii) classification as an original research article, and (iii) studies examining polytrauma populations defined by an Injury Severity Score (ISS) of >15. Studies with populations limited to isolated single-site injuries and cohorts containing pediatric populations were excluded. The literature search and final verification of eligibility were completed in November 2025.

### 2.4. Definition of multitrauma

Multitrauma was defined according to the ISS [8]. In accordance with widely accepted standards, studies including patients with ISS of >15 were considered to represent a multitrauma population [9,10]. This threshold is commonly used to classify severe trauma and is associated with markedly increased morbidity and mortality. To ensure methodological consistency and reduce heterogeneity, the ISS > 15 criterion was applied uniformly during both screening and data extraction.

### 2.5. Classification of statistical methods

Statistical methods were categorized based on the 29-category classification system used by Gritti et al. [1] in the cardiothoracic surgery literature, with the addition of machine learning as a new category. This additional category was designated “Other (Machine Learning, Simulation, etc.).” The statistical methods used were conceptually classified into the educational categories of “Introductory,” “Intermediate,” “Advanced,” and “Specialized” in line with the education-based classification framework presented by four major reference sources in the literature [1,6,11,12]. The “Specialized” category was retained only for descriptive completeness and transparency; it was not treated as an independent analytical level in the present study. This classification was defined based on the minimum level of statistical training required for a reader to fully understand the relevant methods:

- **Basic level:** Descriptive statistics, t-test, chi-square test, analysis of variance (ANOVA), simple correlation, and simple regression, which can be understood with undergraduate biostatistics training.
- **Intermediate level:** Multivariate regression (logistic, Poisson), survival analyses (Cox, Kaplan–Meier), propensity score methods, receiver operating characteristic (ROC) curve and area under the curve (AUC) analysis, missing data approximations, and sensitivity analysis, which require graduate epidemiology/biostatistics training.
- **Advanced level:** Mixed effects models [linear mixed model (LMM), generalized linear mixed model (GLMM), generalized estimating equation (GEE)], Bayesian statistics, machine learning, generalized additive model (GAM), cubic splines, and advanced modeling techniques, which require advanced graduate training.
- **Specialized level:** Limited methods used in specific clinical study designs, such as noninferiority trials or cost/benefit analyses.

Methods falling within the “Specialized” category were analytically incorporated into the “Advanced” level in this study, and all subsequent analyses, tables, and cumulative interpretability assessments were prepared or conducted using the three-level framework of “Introductory,” “Intermediate,” and “Advanced.” This framework shows over 90% agreement with the classification systems proposed by Gritti et al. [1], Tazik et al. [11], Ramoska et al. [6], and Raj and Thulasingam [12].

### 2.6. Article screening and elimination process

Multitrauma articles published between January 2018 and November 2025 were retrieved from the WoS database using the predefined search strategy. Articles were sorted in descending order according to WoS citation metrics. A total of 583 articles were screened to identify the 100 most-cited studies that fulfilled all inclusion criteria.

Exclusion criteria were applied as follows: 1) studies including pediatric populations (n = 126); 2) trauma studies not meeting the ISS-based multitrauma definition, such as isolated organ injuries, mental health-focused trauma research, sports injuries, or e-scooter accidents (n = 109); and 3) nontrauma studies (n = 248). The screening process was terminated once the 100th eligible article was identified. The overall exclusion rate was 82.9% (483/583) and the final sample represents the multitrauma studies with the highest citation impact addressing adult multitrauma patients with ISS of >15.

### 2.7. Ethical approval

This study was based solely on the methodological analysis of published literature and did not involve direct patient data. The study protocol was reviewed and approved by the local institutional ethics committee (AEŞH-BADEK-2024-382) and was conducted in accordance with the principles of the Declaration of Helsinki.

### 2.8. Statistical analysis

All statistical analyses were performed using IBM SPSS Statistics 23.0 (IBM Corp., Armonk, NY, USA) and R Studio 4.4.3 (R Foundation for Statistical Computing, Vienna, Austria). Descriptive statistics were reported as mean ± standard deviation (SD), median, and interquartile range (IQR), as appropriate. Categorical variables were summarized as frequencies and percentages.

Between-group comparisons were conducted using the Mann–Whitney U test or Kruskal–Wallis test for continuous variables, depending on the number of groups and distributional assumptions. Categorical variables were compared using the chi-square test or Fisher exact test as appropriate. A two-sided value of p < 0.05 was considered statistically significant.

Cumulative interpretability analysis was performed according to methodology previously described in the literature [1,6,11,12]. Starting from a baseline of no prior statistical training, we sequentially added each competency level (Introductory, Intermediate, Advanced) and calculated the proportion of articles that could be fully understood at each step. An article was defined as “fully interpretable” once all statistical methods that it contained, up to and including the most complex technique used, were included within the reader’s assumed training level.

## 3. Results

### 3.1. General descriptive findings

A total of 100 articles were included in the analysis (Supplementary Table 1). Across these studies, 1210 statistical procedures were identified within 30 distinct categories. The mean number of statistical methods per article was 12.1 ± 3.8 (range: 5–25; median: 12.0).

All articles used at least one descriptive statistical method and none relied solely on descriptive statistics. All studies explicitly reported the number of participants (n), and percentage calculations were used in 99% of articles. The use of mean and SD was reported in 77% and 72% of studies, respectively, whereas median and IQR values were reported in 79% and 75%. Standard error of the mean (SEM) was rarely used (6%) (Supplementary Table 2).

### 3.2. Frequency of statistical categories

The most frequently used statistical categories were contingency tables (82%, n = 82), epidemiologic statistics (72%, n = 72), and nonparametric tests (63%, n = 63). Among advanced statistical methods, multiple regression was used in 56% (n = 56), missing data methods in 52% (n = 52), and t-tests in 49% (n = 49). Less commonly used approaches included survival analyses (16%, n = 16), ROC analyses (13%, n = 13), and propensity score techniques (12%, n = 12) (Table 1).

### 3.3. Publication characteristics

#### 3.3.1. Publication year and journal distribution

The distribution of publication years showed that the most articles were published in 2018 (n = 37, 37%), followed by 2019 (n = 21, 21%), 2020 (n = 15, 15%), 2021 (n = 15, 15%), 2022 (n = 7, 7%), and 2023 (n = 5, 5%) (Supplementary Table 3).

The 100 analyzed articles were published across 49 different journals. The journals with the highest numbers of publications were *Journal of Trauma and Acute Care Surgery* (n = 15, 15%), *JAMA Surgery* (n = 10, 10%), *European Journal of Trauma and Emergency Surgery* (n = 4, 4%), *Injury* (n = 4, 4%), *Scandinavian Journal of Trauma, Resuscitation and Emergency Medicine* (n = 4, 4%), and *World Journal of Emergency Surgery* (n = 4, 4%) (Supplementary Table 4).

#### 3.3.2. Journal quality indicators

According to the WoS quartile classification, 39% (n = 39) of the articles were published in Q1 journals, 35% (n = 35) in Q2 journals, 20% (n = 20) in Q3 journals, and 6% (n = 6) in Q4 journals. The mean IF of the journals was 5.54 ± 6.86, with a median of 3.38 and a range of 0.55–59.10 (Supplementary Table 5).

### 3.4. Study design and methodological characteristics

#### 3.4.1. Study design

Most of the included studies were retrospective in design (69%, n = 69), while prospective studies accounted for 26% (n = 26). Five studies (5%) were conducted as randomized controlled trials (RCTs) (Supplementary Table 6).

#### 3.4.2. Power and sample size calculations and population weighting

The use of power and sample size calculations differed significantly across study designs (p < 0.001). Power and/or sample size calculations were reported in 80% of RCTs (n = 4/5) but were far less common in prospective studies (11.5%, n = 3/26) and retrospective studies (10.1%, n = 7/69).

Population weighting, defined as applying statistical weights to adjust the sampling structure or representativeness for large datasets, was used exclusively in retrospective studies (13.0%; n = 9/69) (Supplementary Table 7).

### 3.5. Thematic analysis of primary outcomes

Most studies primarily focused on mortality-related outcomes. Other frequently examined primary outcomes included survival indicators, transfusion and hemostasis-related measures, clinical and hospital-based outcomes involving intensive care and emergency department processes, epidemiologic assessments and risk factors, coagulation and biological parameters, and metrics related to system and process performance (Supplementary Table 8).

### 3.6. Statistical software usage

Across the 100 studies, a total of 113 instances of statistical software use were identified, as several articles reported the use of more than one software package. The statistical software used was explicitly named in 90 articles, whereas 10 articles (10%) did not report any specific statistical software.

When examined on a by-instance basis, the most frequently used software was SPSS/IBM SPSS Statistics (29.2%, n = 33/113), followed by Stata/STATA (24.8%, n = 28/113), R/RStudio (11.5%, n = 13/113), SAS (9.7%, n = 11/113), and GraphPad/GraphPad Prism (5.3%, n = 6/113). Other software packages accounted for 10.6% (n = 12/113) of reported instances (Supplementary Table 9).

### 3.7. Use of graphical visualization methods

Overall, graphical visualizations were present in 72% of the analyzed articles (n = 72), while 28% did not contain any graphical elements. Among all articles, the most frequently used visualization type was bar charts, which appeared in 35% of studies, followed by trend or curve plots (31%) and line charts (12%). More advanced or specialized visualization methods were used less frequently, including Kaplan–Meier curves (9%), ROC curves (7%), box plots (7%), forest plots (4%), GLMM curves (3%), cubic spline trend plots (2%), principal component analysis plots (2%), pie charts (2%), Sankey diagrams (2%), GAM curves (1%), and mosaic plots (1%) (Supplementary Table 10).

### 3.8. Bibliometric indicators

The median IF of the journals in which the studies were published was 3.38, with values ranging from 0.55 to 59.10. The median total citation count of the included articles was 35, with a range of 22 to 289. The median number of authors per article was 8, varying between 2 and 36 (Supplementary Table 11).

### 3.9. Statistical complexity levels

Statistical competency requirements varied substantially across the analyzed articles. As shown in Table 2, only a small proportion of studies were interpretable using introductory-level statistical knowledge, whereas most required intermediate or advanced analytical training.

### 3.10. Cumulative interpretability analysis

**Figure 1:**
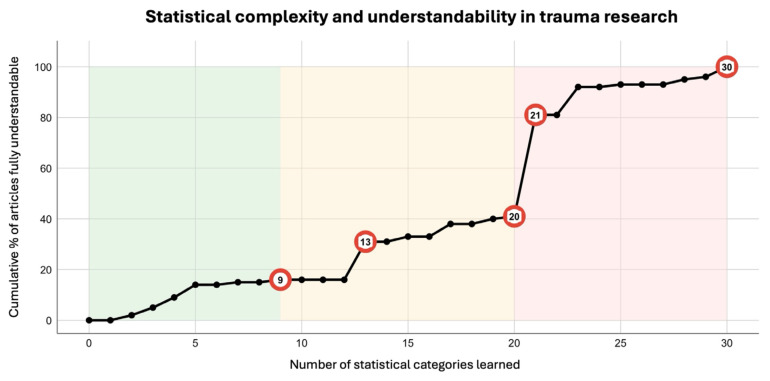
Cumulative percentage of articles fully interpretable by increasing statistical competency.

Cumulative interpretability analysis describes the progressive increase in the proportion of articles that become fully interpretable as additional statistical competencies are acquired. Based on the analytical framework applied, familiarity with descriptive statistics and basic bivariate tests (e.g., t-tests and chi-square analyses) corresponded to full interpretability of approximately 16% of the included articles. When competencies related to multivariable regression and survival analyses such as logistic regression, Cox regression, and Kaplan–Meier methods were incorporated, the cumulative proportion of interpretable articles increased to about 40%. A further marked increase was observed with the inclusion of missing data methodologies, including complete case analysis and multiple imputation, after which cumulative interpretability reached approximately 80%. Full interpretability of all included articles (100%) was observed only after the analytical framework was extended to encompass advanced statistical approaches, including noninferiority analyses, ROC/AUC evaluations, resampling and multivariate techniques, Bayesian methods, and machine learning-based analyses (Figure 1; Table 3).

Table 2 presents an article-level classification based on the highest statistical competency required to interpret each study in its entirety, whereas Table 3 reflects method-level cumulative interpretability analysis, demonstrating how progressive acquisition of statistical knowledge increases the proportion of articles that become fully interpretable.

To illustrate the cumulative interpretability framework, consider a hypothetical set of 10 multitrauma articles. Two articles report only descriptive statistics and bivariate analyses (e.g., chi-square tests) and do not include any multivariable modeling. A reader with introductory-level statistical knowledge would be able to fully interpret only these two articles. Six additional articles include logistic regression analyses in addition to descriptive and bivariate statistics. Although these articles contain basic analyses, the presence of logistic regression means that a reader who lacks regression training cannot fully interpret these studies. Therefore, without intermediate-level statistical competency, these six articles remain not fully interpretable. The remaining two articles apply logistic regression together with missing data methods such as multiple imputation. Consequently, once the reader acquires intermediate-level knowledge including an understanding of multivariable regression, a total of eight articles become fully interpretable. Full interpretability of all ten articles is achieved only when the reader also possesses competency in missing data methodology. This example reflects the cumulative interpretability principle used in this study: an article is considered fully interpretable only when the reader masters all statistical methods applied within that article, and interpretability increases stepwise as additional methodological competencies are acquired.

### 3.11. Relationship between methodological characteristics and journal impact factor

Journal IFs differed across several methodological characteristics. Studies requiring advanced-level statistical training were published in journals with higher IFs compared to those requiring introductory or intermediate-level knowledge. Articles that reported an explicit approach to handling missing data were also associated with higher journal IFs than those that did not.

**Figure 2:**
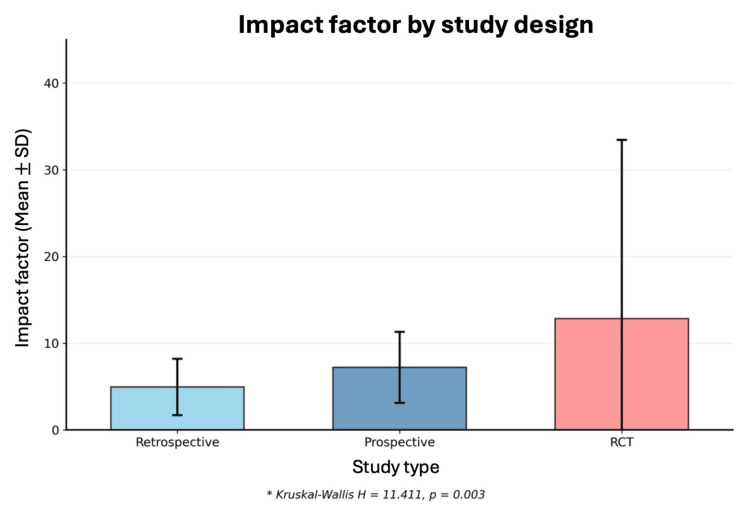
Impact factor by study design. Randomized controlled trials (RCTs) were published in significantly higher-impact journals.

**Figure 3:**
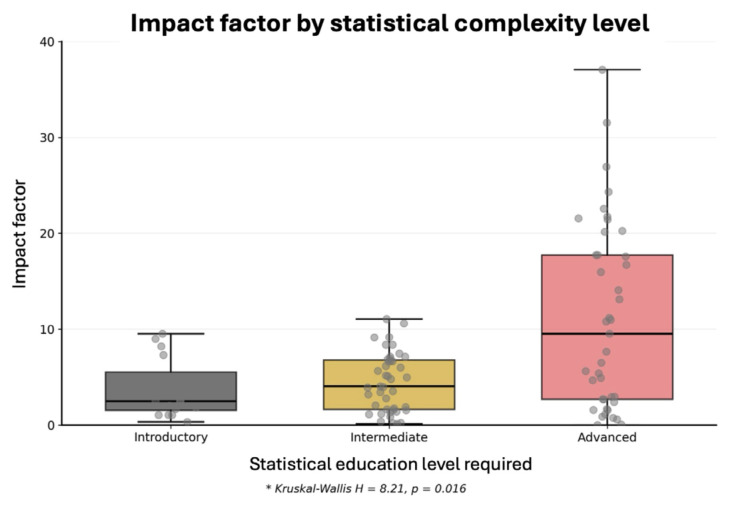
Association between statistical complexity and journal impact factor. The distribution of impact factors across statistical education levels is illustrated. Advanced-level articles were published in significantly higher-impact journals.

In addition, the use and the complexity of graphical visualization methods were associated with journal IFs, with studies employing intermediate or advanced graphical approaches appearing in higher-impact journals compared to articles without graphics or with only basic graphics. Finally, journal IFs varied by study design, with RCTs being published in higher-impact journals than prospective or retrospective studies (Table 4). The distribution and accessibility of each statistical method category are presented in Table 3 and visualized in Figures 2 and 3.

### 3.12. Effect size reporting and mortality-related outcome patterns in multitrauma studies

When the statistical competency level required for each article was cross-tabulated with the presence of mortality or survival outcomes, mortality-related endpoints were most frequently observed in studies requiring intermediate-level statistical training (57.6%, n = 34). Articles requiring advanced-level training accounted for 32.2% of mortality-focused studies (n = 19), whereas only 10.2% (n = 6) of mortality-related articles fell within the introductory competency level. Overall, 59% of the included articles reported mortality or survival outcomes (Supplementary Table 12).

Across the 100 articles, the reporting of effect size increased over time. In 2018, 59.5% of the studies (n = 22/37) reported an effect size, whereas this proportion was 66.7% in 2019 (n = 14/21) and 60.0% in 2020 (n = 9/15). A marked rise was observed in the subsequent years, with effect size reported in 86.7% of studies published in 2021 (n = 13/15) and in all studies published in both 2022 (n = 7/7) and 2023 (n = 5/5). Overall, effect size measures were reported in 70% of all included articles (Supplementary Table 13).

A comprehensive inventory of the distinct statistical techniques identified, along with their detailed classification references and distribution by competency level, is provided in Supplementary Tables 14 and 15.

## 4. Discussion

### 4.1. Principal findings

The most fundamental finding of this study is that the vast majority of the most frequently cited multitrauma articles published in the post-2018 ATLS update period require a level of methodological expertise that exceeds the basic biostatistical knowledge typically acquired during standard undergraduate medical training. Our detailed analysis revealed that only 8% of the examined articles could be fully understood using introductory-level statistical knowledge, whereas the remaining 92% required intermediate or advanced statistical expertise. This indicates a substantial methodological gap between the scientific literature and the clinicians who are expected to interpret and apply this evidence in practice. Such a gap may complicate the critical appraisal of contemporary studies and may pose challenges for the effective interpretation and contextualization of research findings, particularly for early-career clinicians and researchers. Consequently, this gap may represent an area of vulnerability in evidence appraisal.

### 4.2. Impact of statistical literacy deficits on patient safety

One potential concern highlighted by our findings is the possibility that methodologically rigorous but analytically complex studies may not always be fully or accurately interpreted by clinicians who are expected to translate this evidence into clinical practice. In emergency medicine, where decision-making is often rapid and high-stakes, limited familiarity with complex analytical approaches may, in some circumstances, increase the likelihood of misinterpretation. For example, a clinician who misinterprets the results of a multivariable regression model or the clinical significance of a p-value may adopt a treatment that is statistically significant but clinically ineffective. Conversely, a finding that is highly valuable clinically may be overlooked because the clinician does not fully understand the complex analysis from which it was derived. Although the present study does not assess clinical decisions or patient outcomes directly, this interpretive gap between increasingly complex statistical analyses and everyday clinical practice may represent a latent vulnerability in evidence-based care.

### 4.3. Position of the findings within the literature and historical perspective

The methodological complexity observed in the trauma literature is consistent with trends documented across surgical disciplines. Gritti et al. reported that more than 80% of cardiothoracic surgery articles require advanced statistical knowledge [1]. Similarly, Kurichi and Sonnad documented a progressive increase in statistical complexity over an 18-year period in surgical journals [2]. The finding in this study that one out of every three analyzed articles employed advanced-level methods reflects the inherent complexity of this field. Multiple injury patterns, comorbidity burden, heterogeneous treatment protocols, and time-dependent intervention effects in trauma patients often render classical regression models insufficient. In this body of trauma literature, in which such complexity is steadily increasing, it appears that clinicians must inevitably go beyond traditional statistical training in order to function not only as readers but also as researchers. Otherwise, the trauma literature risks evolving into a body of work that can be understood only by those with advanced statistical knowledge.

### 4.4. Relationship between methodological depth and publication impact

This study has identified a significant association between the level of methodological complexity and journal IF in the multitrauma literature. Studies employing advanced statistical methods were, on average, associated with publication in higher-impact journals compared to those using basic or intermediate approaches. Similar associations were reported by Gritti et al. [1], who concluded that the use of more complex statistical methods in the cardiothoracic surgery literature was more frequently observed in higher-impact journals.

In addition, the observed association between the use of contemporary approaches to missing data and higher journal IFs [13] suggests that transparent and methodologically rigorous analytical practices may be more common in journals with greater scientific visibility. However, these findings should be interpreted as associative rather than causal, as journal IF is influenced by multiple editorial, disciplinary, and temporal factors beyond statistical methodology alone.

The higher IF values observed among RCTs suggest that associations between study design characteristics and journal IF may coexist alongside analytical complexity [14,15]. In addition, the observation that studies employing intermediate-level graphical methods were published in higher-impact journals may reflect differences in the presentation and communication of analytical results rather than visualization complexity alone. These findings should be interpreted cautiously as being associative, as neither study design nor graphical complexity was evaluated as a direct determinant of methodological quality.

Moreover, the observed increase in effect size reporting after 2021 may reflect a growing emphasis within the trauma literature on measures of clinical relevance alongside traditional inference based on p-values [16]. This trend suggests that effect sizes are being reported more frequently in more recent publications, particularly in journals with higher IFs, consistent with the broader methodological recommendations in the literature [17].

Taken together, these observations indicate that studies published in higher-impact journals tend to exhibit greater statistical complexity. While statistical complexity should not be equated with methodological rigor or appropriateness, these findings highlight the increasing importance of statistical literacy for researchers and clinicians engaging with contemporary trauma research.

### 4.5. Advanced methods: necessity versus choice

Trauma data frequently include repeated measurements (time-varying physiological parameters), hierarchical structures (patients originating from different centers), and competing outcomes (e.g., discharge versus death). These data characteristics may not be optimally addressed using classical regression approaches alone. Prior studies including the use of the GEE to model time-dependent effects of blood transfusion [18], the application of the GLMM to account for within-patient variability in severe traumatic brain injury [19], and the comparison of competing risks models with Kaplan–Meier methods in the presence of competing outcomes [20] illustrate situations in which advanced analytical approaches can offer more appropriate or informative insights. Collectively, these examples suggest that, in certain trauma research contexts, analytical approaches beyond the introductory level may be warranted.

A key finding of our study is that intermediate and advanced statistical methods are more frequently observed in higher-impact multitrauma journals. This observation aligns with a broader discussion in the scientific literature. Some authors have described this phenomenon as a “statistical arms race,” reflecting increasing expectations for methodological sophistication in published research [21]. It has been suggested that editorial and peer-review processes may, in certain contexts, place strong emphasis on analytical complexity, which could influence authors’ methodological choices, even when simpler approaches might be sufficient. In addition, the perception that more “complex” manuscripts are of “higher quality,” combined with the desire to increase the odds of acceptance by high-impact journals, may be viewed as an important factor contributing to increasing methodological complexity. As noted by Thiese et al. [22], in some cases the choice of statistical methods may be shaped not by scientific rationale but by the intention to reach a desired outcome.

At the same time, a “knowledge deficit” may exist among clinicians and even among researchers. As emphasized by Gritti et al. [1], advances in statistical analysis have not always been accompanied by equivalent improvements in the ability of healthcare professionals to understand and critically interpret increasingly complex analytical approaches. This imbalance may, in some cases, contribute to what has been described as a “toolbox fallacy” [4,23], whereby the availability of powerful statistical software encourages the use of more complex or visually impressive methods, even when simpler approaches would be sufficient. Under such circumstances, there is a risk that, rather than selecting the most appropriate method for a given research question, investigators may adopt a form of copy-and-paste methodology, applying the analytical techniques commonly used in prior high-impact studies without fully appreciating their assumptions or limitations. As also noted by Thiese et al. [22], a substantial proportion of statistical errors may arise from underlying gaps in methodological training and from limited access to, or engagement with, specialized statistical expertise.

### 4.6. In-depth analysis of specific findings

The predominance of intermediate and advanced statistical methods in studies examining mortality or survival as primary outcomes may stem from the structural complexity of trauma data. Because of time-dependent processes, multiple organ failures, physiopathological fluctuations, and highly interrelated risk factors, mortality prognosis may not be adequately assessed using simple comparisons; advanced methodologies such as Cox regression, Kaplan–Meier curves, competing risks analyses, propensity score methods, and sophisticated imputation techniques are therefore often required. Our study has demonstrated that only 10% of articles including mortality outcomes were interpretable using introductory-level statistics, whereas 90% appeared to require intermediate or advanced knowledge. This may point to a clinically relevant concern: insufficient statistical literacy could potentially contribute to misinterpretation of mortality risk and may affect the appropriate application of evidence-based algorithms. Modern trauma data increasingly suggest that clinicians are required not only to be skilled physicians but also to function as informed interpreters of complex data.

In the literature, Raj and Thulasingam [12] suggested that the use of inappropriate statistical methods in surgical studies may be associated with flawed clinical decision-making, while Held et al. [24] stated that reporting quality is markedly higher in studies where biostatisticians are part of the research team. Particularly in studies employing advanced methods such as the GEE, the GLMM, Bayesian analyses, and machine learning, obtaining professional statistical consultation is widely considered important for minimizing methodological errors and improving the reliability of results.

### 4.7. Practical implications: a dual-strategy framework

Our findings suggest the need for a dual-strategy approach to help address the observed methodological gap, with one strategy focused on short-term supportive measures for current clinicians and another aimed at longer-term systemic solutions for future generations.

#### 4.7.1. Application of a methodological filter in evidence appraisal

**Figure 4:**
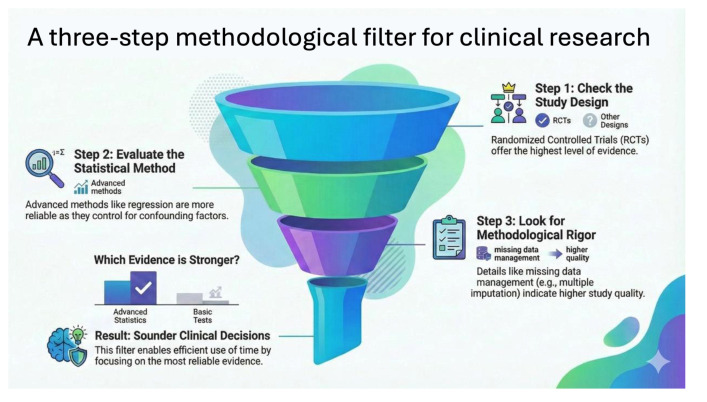
A three-step methodological filter for clinical research.

For clinicians navigating high-impact trauma literature without advanced statistical training, we propose a practical three-step “methodological filter” that may serve as an immediate supportive framework to reduce the risk of misinterpretation of complex analyses (Figure 4). This filter is intended to facilitate real-time critical appraisal and may assist clinicians in evidence evaluation without necessitating extensive additional formal statistical training:

- **Examine the study design:** RCTs are generally considered to provide the strongest evidence. If the study is retrospective or prospective, additional caution may be warranted when interpreting causal claims.
- **Evaluate the level of statistical methods used:** If only basic tests (e.g., t-test, chi-square) are used, confounding factors may not have been fully controlled. Intermediate methods such as logistic regression, Cox analyses, or propensity score methods may indicate greater methodological robustness. Advanced methods such as the GEE or GLMM may suggest an analytical approach appropriate for the complexity of the data and often reflect collaboration with a statistician.
- **Look for indicators of methodological rigor:** Mention of the lack of modern data approaches such as multiple imputation may serve as an important marker that can strengthen the study’s reliability.

This methodological filter may provide clinicians with a practical tool for identifying reliable articles, using time efficiently, and supporting clinical decisions with sound scientific evidence. Like a protective shield, it can function as an immediate defense mechanism that may help current practitioners navigate complex literature more safely while developing deeper expertise. This framework may represent a more comprehensive quality assessment method compared to relying solely on journal IFs. However, the effectiveness of this approach in clinical practice needs to be validated through prospective studies and should be tested across different trauma centers.

#### 4.7.2. Systemic solution: a phased statistical curriculum as methodological immunization

While the proposed methodological filter provides immediate protection, closing the methodological gap at its source may require reform in medical education through a systemic “immunization” that aims to reduce risk rather than treating the symptoms alone. Our findings suggest that statistical literacy is increasingly no longer optional but has become progressively more central to clinical competence, analogous to vaccination, which prevents disease rather than treating symptoms. Similarly, our proposed dual approach may help support both immediate patient safety and the long-term sustainability of evidence-based trauma practice.

**Figure 5:**
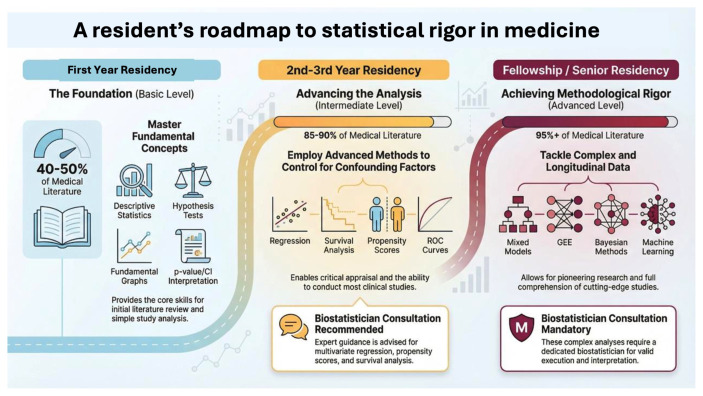
A resident’s roadmap to statistical rigor in medicine.

We propose a phased statistical curriculum aligned with competency-based medical education principles, functioning like stepwise immunization to build immunity gradually [25,26]. In this model, descriptive statistics and simple hypothesis tests could be taught during the first year of residency, while regression analyses, survival methods, and intermediate-level techniques such as propensity score methods may be added in the second and third years. For physicians pursuing research, advanced methods such as the GLMM, GEE, and Bayesian analyses, together with modern approaches to missing data, could be introduced, and collaboration with biostatisticians may be considered essential. Indeed, our findings suggest that biostatistician involvement is associated with improved reliability and reporting quality in studies using advanced analyses (Figure 5) [24].

This approach is not intended to be merely a theoretical proposal; evidence of the educational need has been repeatedly highlighted in the literature. The finding that 92% of the examined articles require intermediate or advanced statistical methods is consistent with the classical study by Windish et al. [3], who reported that the majority of residents may lack the biostatistical knowledge needed to interpret published research. This insufficiency has been described as one of the primary barriers to evidence-based medical practice. Our model, which is conceptually aligned with the stepwise learning approaches recommended by Cheatham et al. [27] and Enders et al. [28], suggests that approximately 40%–50% of the literature could be understood in the first year, 85%–90% with the acquisition of intermediate-level training, and over 95% once advanced methods are incorporated. Moreover, Held et al. [24] and Delgado-Rodríguez et al. [29] reported that biostatistician involvement is associated with higher methodological rigor and improved reporting quality.

At this point, it is not only clinicians in training who may need to adapt their communication approach. Biostatisticians may also need to reconsider how methods are conveyed. Presenting complex methods in a more simplified, visual, and clinically contextualized way, with what could be described as a “translational biostatistics” approach, may constitute a key step in potentially bringing these two worlds closer together. Ultimately, the combination of standardizing phased statistical education and biostatistician collaboration could be viewed not only as a strategy that may enhance publication quality but as a potentially bidirectional bridge that helps to integrate clinical practice with scientific evidence.

### 4.8. Limitations of the study

Several limitations should be considered when interpreting the findings of this study. First, the literature search was restricted to the WoS Core Collection database; inclusion of additional databases such as PubMed or Scopus may have slightly altered the results. Second, our analysis focused on the top 100 most-cited articles. Although citation count is a widely monitored indicator of impact, this approach may have excluded studies that received fewer citations but were methodologically innovative or clinically valuable. In addition, the necessity for citations to accumulate over time introduces the possibility of “citation lag bias,” particularly for articles published in recent years. Third, only articles published in English were included. This may have introduced language bias by excluding studies conducted in other languages with limited accessibility. Finally, the classification of statistical methods according to educational levels inherently contains some degree of subjectivity. Although formal interrater reliability metrics (e.g., intraclass correlation coefficients) were not calculated, all disagreements were resolved through structured consensus discussions among reviewers, with final classifications agreed upon collectively. This consensus-based approach was employed to enhance internal consistency and align the classifications with previously published educational frameworks in the literature.

## 5. Conclusion

Overall, this study suggests that high-impact multitrauma literature often requires intermediate and advanced statistical methods, pointing to a potential methodological gap between clinical training and scientific evidence. To address this challenge, we propose a dual complementary strategy: short-term support for current clinicians using a practical methodological filter, and a longer-term educational approach involving a phased statistical curriculum in emergency medicine and trauma surgery training. In this way, the clinical decision-making process may be grounded in more reliable evidence and evidence-based medical practice may be further strengthened. This transformation can be interpreted as redefining trauma care as a discipline practiced not only with clinical knowledge but also with increasing methodological competence.

## Supplementary Information





## Figures and Tables

**Table 1:** Ten most frequently used statistical categories.

Rank	Category	Number of articles using the method (%)
1	Contingency tables	82 (82%)
2	Epidemiologic statistics	72 (72%)
3	Nonparametric tests	63 (63%)
4	Multiple regression	56 (56%)
5	Missing data methods	52 (52%)
6	t tests	49 (49%)
7	Simple linear regression	17 (17%)
8	Sensitivity analysis	17 (17%)
9	Survival methods	16 (16%)
10	Analysis of variance	16 (16%)

**Table 2:** Distribution of statistical competency levels required to interpret the articles.

Competency level	Description	Number of articles	Percentage (%)	Cumulative (%)
Introductory	Undergraduate-level basic biostatistics	8	8%	8%
Intermediate	Graduate-level epidemiology/biostatistics	61	61%	69%
Advanced	Advanced graduate or specialized training	31	31%	100%
**Total**	—	100	100%	—

**Table 3:** Categorization of statistical methods.

	Summary of statistical methods used across all journals			
	Method category		Articles using these methods (n,%)	Cumulative proportion of accessible articles (n,%)
1	No statistical methods / descriptive Statistics	n number, percentage, mean, SD, median, IQR, SEM	100 (100%)	0 (0%)
2	t tests	t test	49 (49%)	2 (2%)
3	Contingency tables	Chi-square, Fisher’s exact, McNemar	82 (82%)	5 (5%)
4	Nonparametric tests	Mann–Whitney, Kruskal–Wallis, Hodges–Lehmann Difference	63 (63%)	9 (9%)
5	Epidemiologic statistics	Odds_ratio, RR, HR, IRR, SMR, Cohens_d, CI, Sensitivity, Specificity, PPV, NPV, LR	72 (72%)	14 (14%)
6	Propensity score	Propensity matched, IPW, IPWRA	12 (12%)	14 (14%)
7	Pearson correlation	Correlation analysis	12 (12%)	15 (15%)
8	Simple linear regression	Linear regression, coefficient B	17 (17%)	15 (15%)
9	Analysis of variance	ANOVA, post hoc Conover–Imam DSCF	16 (16%)	16 (16%)
10	Transformation/distribution	Box–Cox transformation	1 (1%)	16 (16%)
11	Nonparametric correlation	Kappa	2 (2%)	16 (16%)
12	Survival methods	Cox regression, Kaplan–Meier, log rank, competing risks, Grays test, likelihood ratio	16 (16%)	16 (16%)
13	Multiple regression	logistic regression, Poisson regression, negative binomial, GLM binomial loglinked, GEE, GAM, cubic splines	56 (56%)	31 (31%)
14	Multiple comparisons	Obrien–Fleming correction, interim analysis	4 (4%)	31 (31%)
15	Adjustment & standardization	Population-weighted analysis	9 (9%)	33 (33%)
16	Multiway tables	—	—	—
17	Power analysis	Power analysis	14 (14%)	38 (38%)
18	Cost–benefit analysis	—	—	—
19	Sensitivity analysis	Sensitivity analysis	17 (17%)	40 (40%)
20	Repeated-measures analysis	LMM, GLMM, NLMEM	7 (7%)	41 (41%)
21	Missing data methods	Pairwise exclusion, multiple imputation, complete case analysis, intention to treat, per protocol, worst case assignment, pooled analysis	52 (52%)	81 (81%)
22	Noninferiority trial	—	—	—
23	Receiver operating characteristic	ROC, AUC	13 (13%)	92 (92%)
24	Resampling	—	—	—
25	Principal component analysis	PERMANOVA	2 (2%)	93 (93%)
26	Cluster analysis	—	—	—
27	Metaaanalysis	—	—	—
28	Genetic/statistical genetics	Bioinformatic method	2 (2%)	95 (95%)
29	Bayesian statistics	Bayesian method	3 (3%)	96 (96%)
30	Others (machine learning/miscellaneous/unclassified)	Evidential reasoning, simulation, machine learning, random forests, precision, F1 score, rate difference decomposition	4 (4%)	100 (100%)

Articles using these methods (n, %): Number and proportion of articles in which the specified statistical method was explicitly used and reported. Accessible articles (n, %): Cumulative number and proportion of articles that become fully interpretable once the reader possesses the statistical competency required for the corresponding method within the cumulative interpretability framework; this value does not represent method prevalence.

SD: Standard deviation; IQR: interquartile range; SEM: standard error of the mean; RR: risk ratio, OR: odds ratio; HR: hazard ratio; IRR: incidence rate ratio; SMR: standardized mortality ratio; CI: confidence interval; PPV: positive predictive value; NPV: negative predictive value; LR: likelihood ratio; IPW: inverse probability weighting; IPWRA: inverse probability weighted regression adjustment; ANOVA: analysis of variance; ROC: receiver operating characteristic; AUC: area under the curve; GEE: generalized estimating equation; GLM: generalized linear model; GAM: generalized additive model; LMM: linear mixed model; GLMM: generalized linear mixed model; NLMEM: nonlinear mixed-effects model; PERMANOVA: permutational multivariate analysis of variance, PCA: principal component analysis.

**Table 4:** Comparison of impact factors across methodological characteristics of studies.

Variable	Category	IF (mean ± SD)	Test statistics	p-value
**Level of statistical education**	Introductory	3.98 ± 3.36	Kruskal–Wallis H = 8.21 ɛ^2^ = 0.064	0.016
	Intermediate	3.97 ± 2.97		
	Advanced	9.03 ± 10.79**		
**Missing data approach**	Absent	4.54 ± 3.84	Mann–Whitney U = 728.50 (Z = −1.996) r = 0.20	0.046
	Exist	**8.22 ± 11.31**		
**Graphic style**	**N**	**IF** **(mean ± SD)**	Kruskal–Wallis H (df = 3) ɛ^2^ = 0.05	**p-value**
No graphic	28 (%28)	4.22 ± 3.77	8.024	0.046
Introductory	46 (%46)	4.27 ± 3.21		
Intermediate	11 (%11)	12.49 ± 16.18**		
Advanced	15 (%15)	6.79 ± 6.01		
**Total**	**100**	5.53 ± 6.85		
**Study Type**	**N**	**IF** **(Mean ± SD)**	Kruskal–Wallis H (df = 2) ɛ^2^ = 0.1	
Retrospective	69 (%69)	4.56 ± 4.15	11.411	0.003
Prospective	26 (%26)	5.56 ± 4.26		
Randomized Controlled Trial	5 (%5)	18.87 ± 22.77**		
**Total**	**100**	5.53 ± 6.85		

** Statistically significant pairwise difference in post hoc analysis.

Post hoc pairwise comparisons were performed using Dunn’s test with Bonferroni correction for multiple comparisons. IF: Impact factor.
