# Supplementary material for: Bridging the methodological gap in multitrauma literature: a competency-based educational framework and clinical appraisal filter
Source: Turk J Med Sci. 2026 Jan 30;56(3):746–60. doi: 10.55730/1300-0144.6208 (PMC13398599; doi:10.55730/1300-0144.6208)
Supplement: Supplementary file 1 [file Supplementary-1Results-.pdf]

Supplementary Results

1. The 100 articles included in the study

In this study, the top 100 most cited articles in the Web of Science–Science Citation Index Expanded (SCIE) that evaluate multitrauma patients were systematically reviewed. The 100 articles (Supplementary Table 1) included in the study are as follows:

Supplementary Table 1. Included Articles for Analysis

No	Article Title
1	Plasma-first resuscitation to treat haemorrhagic shock during emergency ground transportation in an urban area: a randomised trial
2	Resuscitative Endovascular Balloon Occlusion of the Aorta and Resuscitative Thoracotomy in Select Patients with Hemorrhagic Shock: Early Results from the AAST AORTA Registry
3	Use of Combat Casualty Care Data to Assess the US Military Trauma System During the Afghanistan and Iraq Conflicts, 2001–2017
4	Association of Prehospital Time to In-Hospital Trauma Mortality in a Physician-Staffed Emergency Medicine System
5	Safety Profile and Impact of Low-Titer Group O Whole Blood for Emergency Use in Trauma
6	Factors Associated With Long-Term Outcomes After Injury (FORTE Study)
7	Use of Resuscitative Endovascular Balloon Occlusion of the Aorta for Proximal Aortic Control in Patients With Severe Hemorrhage and Arrest
8	Pre-hospital Resuscitative Endovascular Balloon Occlusion of the Aorta (REBOA) for Exsanguinating Pelvic Haemorrhage
9	Emergency Department REBOA in Trauma Patients With Exsanguinating Hemorrhage (UK-REBOA RCT)
10	Increased Firearm Injury During the COVID-19 Pandemic: A Hidden Urban Burden
11	Prehospital Whole Blood Reduces Early Mortality in Patients with Hemorrhagic Shock

12	Accuracy of Shock Index versus ABC Score to Predict Need for Massive Transfusion in Trauma Patients
13	Effect of Low-Dose Supplementation of Arginine Vasopressin on Need for Blood Product Transfusions in Patients With Trauma and Hemorrhagic Shock
14	Early fibrinogen concentrate therapy for major haemorrhage in trauma (E-FIT 1)
15	Prehospital tourniquet use in penetrating extremity trauma
16	Incidence and severity of electric scooter related injuries after introduction of an urban rental programme in Vienna
17	Recent opioid use and fall-related injury among older patients with trauma
18	Association of Whole Blood With Survival Among Patients Presenting With Severe Hemorrhage in US and Canadian Adult Civilian Trauma Centers
19	The electric scooter: A surging new mode of transportation that comes with risk to riders
20	An analysis of casualties presenting to military emergency departments in Iraq and Afghanistan
21	Noncompliance with ACS-COT full trauma team activation criteria is associated with undertriage deaths
22	The clinical utility of shock index to predict the need for blood transfusion and outcomes in trauma
23	Is there an association between female gender and outcome in severe trauma? A multi-center analysis in the Netherlands
24	Impact of time to repair on outcomes in patients with lower extremity arterial injuries
25	Efficacy of prehospital administration of fibrinogen concentrate in trauma patients bleeding or presumed to bleed (FIinTIC)
26	Epidemiology of trauma presentations during COVID-19 level 4 lockdown

27	Analysis of risk factors in thoracic trauma patients with a comparison of a modern trauma centre: a mono-centre study
28	Analysis of Prehospital Scene Times and Interventions on Mortality Outcomes in a National Cohort of Penetrating and Blunt Trauma Patients
29	Telemedicine Use Decreases Rural Emergency Department Length of Stay for Transferred North Dakota Trauma Patients
30	Old Age With a Traumatic Mechanism of Injury Should Be a Trauma Team Activation Criterion
31	Optimal Timing of Initiation of Thromboprophylaxis in Spine Trauma Managed Operatively: A Nationwide Propensity-Matched Analysis
32	Optimisation of the dosage of tranexamic acid in trauma patients with population pharmacokinetic analysis
33	Evidential Reasoning Rule-Based Decision Support System for Predicting ICU Admission and In-Hospital Death of Trauma
34	Trauma models to identify major trauma and mortality in the pre-hospital setting
35	Use of Vasopressor Increases the Risk of Mortality in Traumatic Hemorrhagic Shock
36	Zone 1 Endovascular Balloon Occlusion of the Aorta vs Resuscitative Thoracotomy for Patient Resuscitation After Severe Hemorrhagic Shock
37	Timing of repair of blunt thoracic aortic injuries in the thoracic endovascular aortic repair era
38	Profile of trauma mortality and trauma care resources at rural emergency departments and urban trauma centres in Quebec
39	Electric Scooter Orthopaedic Injury Demographics at an Urban Level I Trauma Center
40	Resuscitative endovascular balloon of the aorta is feasible in penetrating chest trauma with major hemorrhage: Proposal of a new institutional deployment algorithm

41	Trends and predictors of mortality in unstable pelvic ring fracture: a 10-year experience with a multidisciplinary institutional protocol
42	Cycling injuries and alcohol
43	Reduction in Mortality Rates of Postinjury Multiple Organ Dysfunction Syndrome: A Prospective Population-Based Cohort Study
44	Association Between Hemorrhage Control Interventions and Mortality in US Trauma Patients With Hemodynamically Unstable Pelvic Fractures
45	Impact of comorbidities on the prognoses of trauma patients: Analysis of a hospital-based trauma registry database
46	Falls from height: A retrospective analysis
47	Emergency Resuscitative Thoracotomy: A Nationwide Analysis of Outcomes and Predictors of Futility
48	Analysis of the medical response to November 2015 Paris terrorist attacks: resource utilization according to the cause of injury
49	Hemostasis as soon as possible? The role of the time to angioembolization in the management of pelvic fracture
50	The Reverse Shock Index Multiplied by Glasgow Coma Scale Score (rSIG) and Prediction of Mortality Outcome in Adult Trauma Patients
51	Penetrating injuries in Germany – epidemiology, management and outcome: an analysis based on the TraumaRegister DGU®
52	The Gut Microbiome Distinguishes Mortality in Trauma Patients Upon Admission to the Emergency Department
53	Resuscitative Endovascular Balloon Occlusion of the Aorta: Implementation and Preliminary Results at an Academic Level I Trauma Center
54	Injury severity score as a predictor of mortality in adult trauma patients by injury mechanism types in the United States

55	Association Between Ionized Calcium Concentrations and Mortality in Trauma Patients Requiring Massive Transfusion Protocol Activation
56	Injury patterns and risk factors for orthopaedic trauma from snowboarding and skiing: a national perspective
57	Circulation first – the time has come to question the sequencing of care in the ABCs of trauma
58	Temporal Changes in REBOA Utilization Practices Are Associated With Increased Survival: An Analysis of the AORTA Registry
59	Fresh Frozen Plasma-to-Packed Red Blood Cell Ratio and Mortality in Traumatic Hemorrhage
60	Traumatic spinal cord injury in Victoria, 2007–2016
61	Resuscitative endovascular balloon occlusion of the aorta in trauma patients with haemorrhagic shock
62	Association Between Implementation of a Geriatric Trauma Clinical Pathway and Changes in Rates of Delirium
63	Single institution trial comparing whole blood vs component therapy
64	Revisiting traumatic cardiac arrest: should CPR be initiated?
65	An Analysis of Presentation, Pattern and Outcome of Chest Trauma Patients at an Urban Level 1 Trauma Center
66	It is time for a change in the management of elderly severely injured patients! An analysis of 126,015 patients from the TraumaRegister DGU®
67	Towards patient-specific management of trauma hemorrhage: the effect of resuscitation therapy on parameters of thromboelastometry
68	Use of the STTGMA tool to risk stratify 1-year functional outcomes and mortality in middle-aged and geriatric trauma patients

69	High variation in trauma center ED–ED transfers: examination of overtriage using the National Trauma Data Bank
70	Questioning dogma: does a GCS of 8 require intubation?
71	A prospective study in severely injured patients reveals an altered gut microbiome is associated with transfusion volume
72	Outcomes After Massive Transfusion in Trauma Patients: Variability Among Trauma Centers
73	Severely Injured Trauma Patients With Admission Hyperfibrinolysis; Is There A Role Of Tranexemic Acid?
74	Partial occlusion, conversion from thoracotomy, undelayed but shorter occlusion: REBOA strategy in Japan
75	An Eastern Association for the Surgery of Trauma multicenter trial examining prehospital procedures in penetrating trauma patients
76	The reports of my death are greatly exaggerated (Ultramassive transfusion)
77	Impact of lockdown during the COVID-19 pandemic on number of patients and patterns of injuries at a level I trauma center
78	Impact of the COVID-19 shutdown on orthopedic trauma numbers and patterns
79	The role of prehospital ultrasound in reducing time to definitive care in abdominal trauma patients with moderate to severe liver and spleen injuries
80	Trauma Coagulopathy and Its Outcomes
81	Systemic Hyperfibrinolysis after Trauma: Targeted Proteomic Analysis
82	Trauma-induced disturbances in ionized calcium levels correlate parabolically with coagulopathy, transfusion, and mortality: a multicentre cohort analysis
83	Patterns and outcomes of zone 3 REBOA use in the management of severe pelvic fractures

84	Feasibility and accuracy of ED frailty identification in older trauma patients: a prospective multi-centre study
85	Epidemiology of adult rib fracture and factors associated with surgical fixation
86	Performance of Modified Early Warning Score (MEWS) and CRAMS score in trauma severity and mortality prediction
87	Hemodynamic Effects of Propofol for Induction of Rapid Sequence Intubation in Traumatically Injured Patients
88	Risk factors and outcomes of unrecognised endobronchial intubation in major trauma patients
89	Platelet Dysfunction in Trauma-Induced Coagulopathy
90	Helicopter Versus Ground Transport in Trauma Patients
91	Reverse Shock Index × GCS (rSIG) as a Predictor of Massive Transfusion
92	A multicenter prospective validation study on disseminated intravascular coagulation in trauma-induced coagulopathy
93	Are on-scene blood transfusions by a helicopter emergency medical service beneficial in trauma care?
94	The impact of hypothermia on outcomes in massively transfused patients
95	The use of ABC score in activation of massive transfusion: The yin and the yang
96	Association of Changes in Antithrombin Activity Over Time With Responsiveness to Enoxaparin Prophylaxis and Risk of Trauma-Related VTE
97	Sex-Based Disparities in Timeliness of Trauma Care and Discharge Disposition
98	Dynamic Effects of Calcium on In Vivo and Ex Vivo Platelet Behavior After Trauma
99	Climbing-Related Injury Among Adults in the United States

100	Bundle of care for blunt chest trauma patients improves analgesia but increases rates of intensive care unit admission

Of the 100 studies, all (100%) reported at least one descriptive statistic. Sample size (*n*) was reported in all studies (100%), and percentage (%) calculations were present in 99% of the articles. Mean and SD were reported in 77% and 72% of the studies, respectively. Median and IQR were used in 79% and 75% of the studies. Standard error of mean (SEM) was rarely reported (6%). (Supplementary Table 2)

Supplementary Table 2. Usage of descriptive statistics (n = 100)

Descriptive statistics	Exist n (%)	Absent n (%)
Number of participants (n)	100 (100.0)	0 (0.0)
Percentage (%)	99 (99.0)	1 (1.0)
Mean value	77 (77.0)	23 (23.0)
Standard deviation (SD)	72 (72.0)	28 (28.0)
Median	79 (79.0)	21 (21.0)
Interquartile range (IQR)	75 (75.0)	25 (25.0)
Standard error of mean (SEM)	6 (6.0)	94 (94.0)
**Any of the descriptive statistics**	**100 (100.0)**	**0 (0.0)**

2. Descriptive findings of the journals

When examining the distribution of the 100 articles included in the study by publication year, the highest number of publications was in 2018 (n=37), followed by 2019 (n=21), 2020 (n=15), 2021 (n=15), 2022 (n=7), and 2023 (n=5). (Supplementary Table 3)

Supplementary Table 3. Distribution of articles by publication year

Publication year	n	%
2018	37	37.0
2019	21	21.0
2020	15	15.0
2021	15	15.0
2022	7	7.0
2023	5	5.0
**Total**	**100**	**100.0**

3. Journals in which articles are published

The included studies were published across a wide range of journals, reflecting the multidisciplinary nature of trauma and emergency medicine research. A total of 49 different journals were identified. The journal with the highest number of publications was Journal of Trauma and Acute Care Surgery (n = 15), followed by JAMA Surgery (n = 10). Four journals published four articles each, including *European Journal of Trauma and Emergency Surgery*, *Injury*, *Scandinavian Journal of Trauma, Resuscitation and Emergency Medicine*, and *World Journal of Emergency Surgery*. Additionally, American Journal of Emergency Medicine, Critical Care, and Journal of Surgical Research each contributed three articles. The remaining journals published one or two articles. (Supplementary Table 4)

Academic Emergency Medicine
American Journal of Emergency Medicine ×3
American Surgeon
Anaesth Crit Care Pain Med
Anaesthesia
Anesthesia & Analgesia
Annals of Surgery
Archives of Orthopaedic and Trauma Surgery
BMC Public Health
BMJ Open
British Journal of Surgery
Canadian Medical Association Journal (CMAJ)
Critical Care ×3
Critical Care Medicine
Emergency Medicine Journal
European Journal of Anaesthesiology

European Journal of Emergency Medicine
European Journal of Trauma and Emergency Surgery ×4
IEEE Transactions on Systems, Man, and Cybernetics: Systems
Indian Journal of Surgery
Injury ×4
Intensive Care Medicine
International Journal of Environmental Research and Public Health
JAMA Network Open
JAMA Surgery ×10
Journal of Orthopaedic Trauma ×2
Journal of Surgical Research ×3
Journal of Thrombosis and Haemostasis ×2
Journal of Trauma and Acute Care Surgery ×15
Journal of Vascular Surgery ×2
Knee Surgery, Sports Traumatology, Arthroscopy
Medicine
Medical Journal of Australia
PeerJ
PLOS ONE
Prehospital Emergency Care
Resuscitation
Scandinavian Journal of Trauma, Resuscitation and Emergency Medicine ×4
SHOCK ×2
Telemedicine and e-Health
The Journal of Emergency Medicine
The Lancet
Traffic Injury Prevention
Transfusion
Wilderness & Environmental Medicine
World Journal of Emergency Medicine
World Journal of Emergency Surgery ×4
Wien Klin Wochenschr

Supplementary Table 4. Journals with the most publications

Journal Name	Article count
Journal of Trauma and Acute Care Surgery	15
JAMA Surgery	10
European Journal of Trauma and Emergency Surgery	4
Injury	4

Scandinavian J. Trauma Resuscitation Emergency Medicine	4
World Journal of Emergency Surgery	4
American Journal of Emergency Medicine	3
Critical Care	3
Journal of Surgical Research	3

The Web of Science (WOS) quartile analysis revealed that 39 studies (39.0%) were published in Q1 journals, followed by 35 studies (35.0%) in Q2 journals. A total of 20 studies (20.0%) were published in Q3 journals, while 6 studies (6.0%) appeared in Q4 journals. Overall, all 100 studies (100.0%) were successfully classified across the four quartile levels. (Supplementary Table 5)

Supplementary Table 5. WOS quartile (Q1–Q4) distribution of journals

**Quartile Levels**	**n**	**%**
Q1	39	39
Q2	35	35
Q3	20	20
Q4	6	6
**Total**	**100**	**100**

A total of 100 studies were included in the analysis. The majority were retrospective in design (n = 69; 69.0%), followed by prospective studies (n = 26; 26.0%). Only five studies were classified as Randomized Controlled Trials (RCTs), accounting for 5.0% of the total. (Supplementary Table 6)

Supplementary Table 6. Distribution of articles by study type

Study Type	n	%
Prospective	26	26.0
Retrospective	69	69.0
Randomized Controlled Trial (RCT)	5	5.0
**Total**	**100**	**100.0**

Power analysis was reported in 14% of the studies, while population weighting was present in 9% of the studies. Among randomized controlled trials (n = 5), power analysis was performed in four studies, whereas population weighting was not reported in any of them. In prospective studies (n = 26), power analysis was performed in three studies and population weighting was not applied. Among retrospective studies (n = 69), seven studies reported a power analysis, and nine studies reported population weighting. (Supplementary Table 7)

Supplementary Table 7. Power analysis and distribution of population weighted status by study type

Variable		RCT	Prospective	Retrospective	p-value
**Population Weighted**	**Applied**	0	0	9	0.132
	**Not Applied**	5	26	60	
**Power Analysis**	**Performed**	4	3	7	0.002*
	**Not Performed**	1	23	62	
**Total**		5	26	69	

**Fisher-Freeman-Halton Exact Test*

The majority of primary outcomes in the studies reviewed focused on mortality measures. In addition, survival indicators, transfusion and hemostasis-related outcomes, clinical/hospital outcomes involving intensive care and emergency department processes, epidemiological assessments and risk factors, coagulopathy and biological parameters, and process and system

performance measures were also found to be among the main focuses of various studies. (Supplementary Table 8)

Supplementary Table 8. Thematic groups of primary outcomes

Category	Outcomes for this group
A. Mortality assessment	28-day mortality; Overall mortality; in-hospital mortality; 24-hour mortality; 30-day mortality; 90-day mortality; short term mortality (ED/6h/hospital); survival to discharge; in-hospital survival; 1-year mortality; mortality
B. Survival	Survival; Functional Outcome
C. Transfusion / Haemostasis / MTP	Massive Transfusion Protocol; total transfusion volume within 48 hours; fibrinogen ≥2 g/L; transfusion need; Tranexamic Acid/pharmacokinetics; FIBTEM MCF; massive transfusion; relations between mortality/transfusion volume; timing of massive transfusion
D. Clinical Course	ICU admission; Lenght of stay ED; delirium; algorithm of REBOA (Clinical Course); postintubation hypotension; endobronchial intubation risk factors; diagnosis of frailty
E. Epidemiology / Risc Factors	Epidemiology; Opioid-Related Disorders; alcohol–head injury relations; Protective Devices & ISS >15; incidens; impact of COVID shutdown
F. Coagulopathy / Microbiota / Proteomic	Trauma induced Coagulopathy; DIC; hyperfibrinolise proteomic differences; changes in the gut microbiota
G. Performance of system & outcome of the process	Door to CT/operation room; resource utilization; overtriage; Trauma Team Activation & age criteria

Across all studies, a total of 113 instances of statistical software use were identified, as several articles reported the use of more than one software package. The statistical software used was explicitly reported in 90 articles, whereas 10 articles (10%) did not report any statistical software.

When examined on an instance basis, the most frequently used software was SPSS (29.2%, n = 33/113), followed by Stata/STATA (24.8%, n = 28/113), R/RStudio (11.5%, n = 13/113), SAS (9.7%, n = 11/113), and GraphPad/GraphPad Prism (5.3%, n = 6/113). Other software packages accounted for 10.6% (n = 12/113) of reported instances (Supplementary Table 9).

Supplementary Table 9. Usage descriptives of statistical softwares

Software	Frequency (n)	%
**SPSS**	33	29.2%
**STATA**	28	24.8%
**R / R Studio**	13	11.5%
**SAS**	11	9.7%
**GraphPad / GraphPad Prism**	6	5.3%
**Others** (Excel, QIIME, STAMP, Monolix, MATLAB, JMP, Statistica, Python, XLSTAT)	12	10.6%
**Unknown**	10	8.8%

**Percentages are calculated based on the total number of reported software instances (n = 113), as some articles reported more than one statistical software.*

4. Usage of graphics

Overall, graphical visualizations were present in 72% of the included articles (n = 72), while 28% of studies did not contain any graphical elements. Among all articles, the most frequently used visualization type was bar charts, which appeared in 35% of studies, followed by trend or curve plots (31%) and line charts (12%). More advanced or specialized visualization methods were used less frequently, including Kaplan–Meier curves (9%), ROC curves (7%), box plots (7%), forest plots (4%), GLMM curves (3%), cubic spline trend plots (2%), PCA plots (2%), pie charts (2%), Sankey diagrams (2%), GAM curves (1%), and mosaic plots (1%). As multiple graphical elements could be used within a single article, the total frequency of graphical types exceeds the number of included studies. (Supplementary Table 10)

Supplementary Table 10. Usage frequencies of graphic types

**Graphical visualization type**	**Articles using the method, n (%)**
Any graphical visualization (≥1)	*72 (72.0)*
No graphical visualization	*28 (28.0)*
	100 (100.0)
Bar chart	35 (35.0)
Trend / curve plot (time-series)	31 (31.0)
Line chart	12 (12.0)
Scatter plot	10 (10.0)
Kaplan–Meier curve	9 (9.0)
ROC curve	7 (7.0)
Box plot	7 (7.0)
Forest plot	4 (4.0)
GLMM curve	3 (3.0)
Cubic spline trend plot	2 (2.0)
Pie chart	2 (2.0)
PCA plot	2 (2.0)
Sankey diagram	2 (2.0)
GAM curve	1 (1.0)
Mosaic plot	1 (1.0)

Table note: *Percentages are calculated on an article basis (n = 100). Individual articles may include more than one type of graphical visualization.*

When examining the Impact Factor values, the average was found to be 5.54, the median 3.38, and the minimum–maximum range varied between 0.55 and 59.10. The total number of authors in the articles averaged 9.32, with a median of 8, and the number of authors ranged from 2 to 36. When evaluating the total number of citations, the average was 50.12, the median was 35.00, and the number of citations ranged from 22 to 289. (Supplementary Table 11)

Supplementary Table 11. Descriptive Statistics of Bibliometric Indicators

Variable	Mean	SD	Median	IQR	Min-Max
Impact Factor (IF)	5.54	6.86	3.38	3.58	0.55 – 59.10
Number of citations	50.12	42.52	35	27	22 – 289
Number of authors	-	-	8	6	2 – 36

6. Effect size reporting and mortality-related outcome patterns in multitrauma studies

The vast majority of studies reporting mortality or survival outcomes (89.8%) required statistical competency beyond the introductory level. (Supplementary Table 12)

Supplementary Table 12. Statistical education level required for the article × Mortality/Survival

Knowledge level	Mortality Exist
Introductory (8)	6 (10.2%)
Intermediate (61)	34 (57.6%)
Advanced (31)	19 (32.2%)
**Total (100)**	**59%**

Across the 100 included articles, effect size reporting demonstrated a clear increasing trend over time. In 2018, effect size measures were reported in 59.5% of studies (n = 22/37), which

increased to 66.7% in 2019 (n = 14/21) and remained comparable in 2020 at 60.0% (n = 9/15). A more pronounced increase was observed from 2021 onward, with effect size reporting present in 86.7% of studies published in 2021 (n = 13/15). Notably, all studies published in 2022 (n = 7/7) and 2023 (n = 5/5) reported effect size measures. Overall, effect sizes were reported in 70% of all included articles, indicating a substantial improvement in reporting practices over time. (Supplementary Table 13)

Supplementary Table 13. Reporting Status of Effect Size by Publication Year

**Publication year**	**Effect size reported n (%)**	**Effect size not reported n (%)**	**Total (n)**
2018	22 (59.5)	15 (40.5)	37
2019	14 (66.7)	7 (33.3)	21
2020	9 (60.0)	6 (40.0)	15
2021	13 (86.7)	2 (13.3)	15
2022	7 (100.0)	0 (0.0)	7
2023	5 (100.0)	0 (0.0)	5
**Total**	**70 (70.0)**	**30 (30.0)**	**100**

Table note: *Percentages are calculated within each publication year.*
