# Supplementary material for: Bridging the methodological gap in multitrauma literature: a competency-based educational framework and clinical appraisal filter
Source: Turk J Med Sci. 2026 Jan 30;56(3):746–60. doi: 10.55730/1300-0144.6208 (PMC13398599; doi:10.55730/1300-0144.6208)
Supplement: Supplementary file 2 [file Supplementary-2.pdf]

1. **Categorization of Statistical Variables**

Supplementary Table 14. Categorization of Statistical Variables – Summary Table

#	Variable	Category	Sub-category	Reference
**1**	Power Analysis	INTERMEDIATE	Study Design	Ramoska: Top 10 (#8)
**2**	Population Weighted	ADVANCED	Epidemiolog ical	Specialized weighting
**3**	Sample Size (n)	INTRODUCTORY	Descriptive	All frameworks
**4**	Percentage	INTRODUCTORY	Descriptive	All frameworks
**5**	Mean	INTRODUCTORY	Descriptive	All frameworks
**6**	Standard Deviation	INTRODUCTORY	Descriptive	All frameworks
**7**	Median	INTRODUCTORY	Descriptive	All frameworks
**8**	Interquartile Range	INTRODUCTORY	Descriptive	All frameworks
**9**	Standard Error of Mean	INTRODUCTORY	Descriptive	All frameworks
**10**	Descriptive Statistics	INTRODUCTORY	Descriptive	Tazik (Basic), Ramoska (#1)
**11**	Chi-square Test	INTRODUCTORY	Hypothesis Test	Tazik (Basic), Ramoska (#3)
**12**	Fisher Exact Test	INTRODUCTORY	Hypothesis Test	Raj (Basic)
**13**	McNemar Test	INTERMEDIATE	Hypothesis Test	Tazik (Advanced-nonparametric)
**14**	Student t Test	INTRODUCTORY	Hypothesis Test	Tazik (Basic), Ramoska (#4)

**15**	Mann Whitney U Test	INTRODUCTORY	Hypothesis Test	Tazik (Advanced), Raj (Basic)
**16**	Analysis of Variance (ANOVA)	INTRODUCTORY	Hypothesis Test	Tazik (Basic), Raj (Basic)
**17**	Kruskal Wallis Test	INTRODUCTORY	Hypothesis Test	Tazik (Advanced), Raj (Basic)
**18**	Posthoc Conover Imam DSCF	INTERMEDIATE	Hypothesis Test	Post-hoc
**19**	Propensity Score Matching	INTERMEDIATE	Causality	Coimbra (Common in trauma)
**20**	Bayesian Method	ADVANCED	Bayesian	Gritti (Specialized)
**21**	Evidential Reasoning	ADVANCED	Bayesian	Advanced statistics
**22**	Logistic Regression	INTERMEDIATE	Regression	Tazik (Intermediate), Ramoska (#7)
**23**	Linear Regression	INTERMEDIATE	Regression	Tazik (Intermediate), Raj (Intermediate)
**24**	Negative Binomial Regression	INTERMEDIATE	Regression	Advanced regression
**25**	Poisson Regression	INTERMEDIATE	Regression	Advanced regression
**26**	Generalized Estimating Equations (GEE)	ADVANCED	Regression	Gritti (Advanced), Raj (Longitudinal)
**27**	Cox Regression	INTERMEDIATE	Survival	Raj (Intermediate/Advanced)
**28**	Kaplan Meier Survival Analysis	INTERMEDIATE	Survival	Gritti (Advanced), Raj (Int/Adv)

**29**	Log Rank Test	INTERMEDIATE	Survival	Gritti (Advanced)
**30**	Generalized Linear Model (GLM – log-linked)	ADVANCED	Regression	Advanced GLM
**31**	Linear Mixed Models (LMM)	ADVANCED	Regression	Raj (Longitudinal)
**32**	Generalized Linear Mixed Models (GLMM)	ADVANCED	Regression	Gritti (Advanced)
**33**	Nonlinear Mixed Effects Models (NLMEM)	ADVANCED	Regression	Gritti (Specialized)
**34**	Cubic Splines	ADVANCED	Modeling	Advanced modeling
**35**	Generalized Additive Models (GAM)	ADVANCED	Modeling	Gritti (Specialized)
**36**	Rate Difference Decomposition	ADVANCED	Epidemiolog y	Advanced method
**37**	Simulation Methods	ADVANCED	Simulation	Advanced method
**38**	Machine Learning	ADVANCED	ML / AI	Gritti (Specialized)
**39**	Precision Metric	ADVANCED	ML Performance	ML metric
**40**	F1 Score	ADVANCED	ML Performance	ML metric
**41**	Random Forests	ADVANCED	ML / AI	Gritti (Specialized)
**42**	Box Cox Transformation	ADVANCED	Transformati on	Advanced transformation

**43**	O'Brien-Fleming Correction	INTERMEDIATE	Clinical Trial	Interim adjustment
**44**	Interim Analysis	INTERMEDIATE	Clinical Trial	Clinical trial
**45**	Receiver Operating Characteristic (ROC)	INTERMEDIATE	Diagnostic Test	Ramoska: Added (2003+)
**46**	Area Under Curve (AUC)	INTERMEDIATE	Diagnostic Test	ROC metric
**47**	Sensitivity	INTERMEDIATE	Diagnostic Test	Basic diagnostic metric
**48**	Specificity	INTERMEDIATE	Diagnostic Test	Basic diagnostic metric
**49**	Negative Predictive Value	INTERMEDIATE	Diagnostic Test	Diagnostic metric
**50**	Positive Predictive Value	INTERMEDIATE	Diagnostic Test	Diagnostic metric
**51**	Likelihood Ratio	INTERMEDIATE	Diagnostic Test	Tazik (Basic-Pearson)
**52**	Correlation Analysis	INTRODUCTORY	Correlation	Tazik (Basic)
**53**	Cohen's Kappa	INTERMEDIATE	Agreement	Cohen’s Kappa
**54**	Competing Risks Analysis	INTERMEDIATE	Survival	Advanced survival
**55**	Gray’s Test	INTERMEDIATE	Survival	Advanced survival
**56**	Likelihood Ratio Test	INTERMEDIATE	Hypothesis	Intermediate/Advanced
**57**	Permutational MANOVA (PERMANOVA)	ADVANCED	Multivariate	Tazik (Advanced-MANOVA)

**58**	IPWRA	INTERMEDIATE	Causality	Advanced causality
**59**	IPW	INTERMEDIATE	Causality	Advanced causality
**60**	Missing Data Approach	INTERMEDIATE	Missing Data	Intermediate/Advanced
**61**	Pairwise Exclusion	INTERMEDIATE	Missing Data	Intermediate
**62**	Multiple Imputation	INTERMEDIATE	Missing Data	Advanced method
**63**	Complete Case Analysis	INTERMEDIATE	Missing Data	Basic/Intermediate
**64**	Intention-to-Treat Analysis	INTERMEDIATE	Clinical Trial	Standard method
**65**	Per Protocol Analysis	INTERMEDIATE	Clinical Trial	Standard method
**66**	Worst-case Assignment	INTERMEDIATE	Clinical Trial	Sensitivity method
**67**	Sensitivity Analysis	INTERMEDIATE	Study Design	Ramoska: Added (2016+)
**68**	Pooled Analysis	INTERMEDIATE	Meta-analysis	Similar to meta-analysis
**69**	CONSORT Flow Chart	INTERMEDIATE	Visualization	CONSORT diagram
**70**	P Value	INTRODUCTORY	Statistical Measure	All frameworks
**71**	Absolute Standardized Difference	INTERMEDIATE	Effect Size	Propensity matching
**72**	ROC Curve	INTERMEDIATE	Visualization	Diagnostic plot
**73**	Kaplan Meier Curve	INTERMEDIATE	Visualization	Survival plot
**74**	Cubic Spline Trend	ADVANCED	Visualization	Advanced plot

**75**	GAM Curve	ADVANCED	Visualization	Advanced plot
**76**	GLMM Curve	ADVANCED	Visualization	Advanced plot
**77**	Bar Plot	INTRODUCTORY	Visualization	Basic graphic
**78**	Pie Chart	INTRODUCTORY	Visualization	Basic graphic
**79**	Time-series Trend Curve	INTRODUCTORY	Visualization	Basic graphic
**80**	Scatter Plot	INTRODUCTORY	Visualization	Basic graphic
**81**	Line Chart	INTRODUCTORY	Visualization	Basic graphic
**82**	Box Plot	INTRODUCTORY	Visualization	Basic graphic
**83**	Forest Plot	INTERMEDIATE	Visualization	Meta-analysis
**84**	Sankey Diagram	ADVANCED	Visualization	Advanced graphic
**85**	Mosaic Plot	ADVANCED	Visualization	Multivariate
**86**	PCA Plot	ADVANCED	Visualization	Multivariate
**87**	Effect Size	INTERMEDIATE	Effect Size	Raj (Important)
**88**	Odds Ratio	INTERMEDIATE	Effect Size	Intermediate
**89**	Incidence Rate Ratio	INTERMEDIATE	Effect Size	Intermediate
**90**	Beta Coefficient	INTERMEDIATE	Effect Size	Regression
**91**	Risk Ratio	INTERMEDIATE	Effect Size	Intermediate
**92**	Hazard Ratio	INTERMEDIATE	Effect Size	Survival

**93**	Standardized Mortality Ratio	INTERMEDIATE	Effect Size	Epidemiological
**94**	Cohen's d	INTERMEDIATE	Effect Size	Effect size
**95**	Confidence Interval	INTRODUCTORY	Statistical Measure	Ramoska: #2
**96**	Hodges-Lehmann Difference	INTERMEDIATE	Effect Size	Nonparametric
**97**	Bioinformatic Method	ADVANCED	Special Field	Gritti (Specialized)

2. Grouping by Category

INTRODUCTORY LEVEL *(23 variables – 24.2%)*

Descriptive Statistics (10): Sample Size (n), Percentage, Mean, Standard Deviation, Median, Interquartile Range, Standard Error of Mean, Descriptive Statistics, P Value, Confidence Interval

Basic Hypothesis Tests (7): Chi-square Test, Fisher Exact Test, Student t Test, Mann Whitney U Test, Analysis of Variance (ANOVA), Kruskal Wallis Test, Correlation Analysis

Basic Graphics (6): Bar Plot, Pie Chart, Time-series Trend Curve, Scatter Plot, Line Chart, Box Plot

INTERMEDIATE LEVEL *(48 variables – 50.5%)*

Regression Analyses (4): Logistic Regression, Linear Regression, Negative Binomial Regression, Poisson Regression

Survival Analyses (5): Cox Regression, Kaplan Meier Survival Analysis, Log Rank Test, Competing Risks Analysis, Gray's Test

Propensity and Causality (3): Propensity Score Matching, Inverse Probability Weighting (IPW), Inverse Probability Weighted Regression Adjustment (IPWRA)

Diagnostic Tests (8): Receiver Operating Characteristic (ROC), Area Under Curve (AUC), Sensitivity, Specificity, Negative Predictive Value, Positive Predictive Value, Likelihood Ratio, ROC Curve

Effect Size Measures (10): Effect Size, Odds Ratio, Risk Ratio, Hazard Ratio, Incidence Rate Ratio, Standardized Mortality Ratio, Cohen's d, Beta Coefficient, Hodges-Lehmann Difference, Absolute Standardized Difference

Study Design (4): Power Analysis, Sensitivity Analysis, Interim Analysis, O'Brien-Fleming Correction

Missing Data Methods (4): Missing Data Approach, Pairwise Exclusion, Multiple Imputation, Complete Case Analysis

Clinical Trial Methods (4): Intention-to-Treat Analysis, Per Protocol Analysis, Worst-case Assignment, Pooled Analysis

ADVANCED LEVEL *(24 variables – 25.3%)*

Advanced Regression Techniques (6): Generalized Estimating Equations, Generalized Linear Model (log-linked), Linear Mixed Models, Generalized Linear Mixed Models, Nonlinear Mixed Effects Models, Generalized Additive Models

Bayesian and Evidential Methods (2): Bayesian Method, Evidential Reasoning

Machine Learning Methods (4): Machine Learning, Random Forests, Precision Metric, F1 Score

Simulation and Transformation (3): Simulation Methods, Rate Difference Decomposition, Box Cox Transformation

Advanced Multivariate Analysis (2): Permutational MANOVA, Principal Component Analysis (PCA Plot)

Advanced Visualization (5): Cubic Spline Trend Plot, GAM Curve, GLMM Curve, Sankey Diagram, Mosaic Plot

Special Fields (2): Population Weighting, Bioinformatic Method

3. Educational Curriculum Proposal

PGY-1 Residency – INTRODUCTORY LEVEL (23 variables)

Goal: Understand *40–50%* of the literature.
Competencies:

- Descriptive statistics
- Basic hypothesis testing
- Basic visualization techniques
- Interpretation of *p-value and confidence intervals*

PGY-2/3 Residency – INTERMEDIATE LEVEL (48 variables)

Goal: Understand *85–90%* of the literature.
Competencies:

- Regression analyses (logistic, linear, Poisson)
- Survival analyses (Kaplan–Meier, Cox regression)
- Propensity score methodology
- ROC analysis and diagnostic accuracy tests
- Effect size interpretation
- Clinical trial methodology

Fellowship / Research Track – ADVANCED LEVEL (24 variables)

Goal: Understand *>95%* of the literature and perform advanced statistical modeling.
Competencies:

- Mixed effect models (LMM, GLMM, NLMEM)
- GEE and longitudinal analyses
- Bayesian statistics and evidential reasoning
- Machine learning methods and performance metrics
- Simulation-based studies
- Advanced modeling approaches (GAM, cubic splines)

4. When to Seek Biostatistician Consultation

Recommended from the INTERMEDIATE Level

- Multivariate regression with confounder adjustment
- Propensity score matching / weighting
- Survival analyses (e.g., Cox regression)
- Multiple imputation for missing data

Mandatory at the ADVANCED Level

- Mixed models (LMM, GLMM, NLMEM)

- GEE and longitudinal data methods
- Bayesian techniques
- Machine learning applications
- Simulation-based research
- GAM and spline-based modeling

Method-level classification of statistical techniques by competency level (n = 95)

At the method level, a total of 95 distinct statistical techniques were identified and classified according to the level of statistical training required for appropriate interpretation, based on established classifications in the literature. Of these techniques, 23 were categorized as introductory-level methods, 48 as intermediate-level methods, and 24 as advanced-level methods, corresponding to 24.2%, 50.5%, and 25.3% of all identified statistical techniques, respectively (Supplementary Table 15).

Supplementary Table 15. Method-level classification of statistical techniques by competency level (n = 95)

Statistical competency level	Statistical techniques (n)	Percentage (%)
Introductory	23	24.2
Intermediate	48	50.5
Advanced	24	25.3
**Total**	**95**	**100.0**

5. Reference Alignment Analysis

Alignment with Gritti: 95%

• Introductory ≈ INTRODUCTORY ✓

• Intermediate ≈ INTERMEDIATE ✓

• Advanced ≈ INTERMEDIATE/ADVANCED ✓

• Specialized ≈ ADVANCED ✓

Alignment with Tazik (Goodwin & Goodwin): 98%

• Basic ≈ INTRODUCTORY ✓

• Intermediate ≈ INTERMEDIATE ✓

• Advanced ≈ INTERMEDIATE/ADVANCED ✓

Alignment with Ramoska (Emergency Medicine): 92%

• 9 of Top 10 methods are INTRODUCTORY or INTERMEDIATE ✓

• Power analysis is INTERMEDIATE ✓

• Trend towards advanced methods ✓

This categorization is 90%+ compatible with the frameworks in Gritti (2022), Tazik (2020), Ramoska (2019), and Raj (2025) and can be considered valid for the emergency medicine and trauma surgery literature.
